# Multi-Scale Attention Network for Vertical Seed Distribution in Soybean Breeding Fields

**DOI:** 10.34133/plantphenomics.0260

**Published:** 2024-11-10

**Authors:** Tang Li, Pieter M. Blok, James Burridge, Akito Kaga, Wei Guo

**Affiliations:** ^1^ Graduate School of Agricultural and Life Sciences, The University of Tokyo, Tokyo, Japan.; ^2^ Institute of Crop Sciences, National Agriculture and Food Research Organization, Tsukuba, Ibaraki, Japan.

## Abstract

The increase in the global population is leading to a doubling of the demand for protein. Soybean (*Glycine max*), a key contributor to global plant-based protein supplies, requires ongoing yield enhancements to keep pace with increasing demand. Precise, on-plant seed counting and localization may catalyze breeding selection of shoot architectures and seed localization patterns related to superior performance in high planting density and contribute to increased yield. Traditional manual counting and localization methods are labor-intensive and prone to error, necessitating more efficient approaches for yield prediction and seed distribution analysis. To solve this, we propose MSANet: a novel deep learning framework tailored for counting and localization of soybean seeds on mature field-grown soy plants. A multi-scale attention map mechanism was applied to maximize model performance in seed counting and localization in soybean breeding fields. We compared our model with a previous state-of-the-art model using the benchmark dataset and an enlarged dataset, including various soybean genotypes. Our model outperforms previous state-of-the-art methods on all datasets across various soybean genotypes on both counting and localization tasks. Furthermore, our model also performed well on in-canopy 360° video, dramatically increasing data collection efficiency. We also propose a technique that enables previously inaccessible insights into the phenotypic and genetic diversity of single plant vertical seed distribution, which may accelerate the breeding process. To accelerate further research in this domain, we have made our dataset and software publicly available: https://github.com/UTokyo-FieldPhenomics-Lab/MSANet.

## Introduction

The world’s population is projected to increase to approximately 8.5 billion by 2030, with an additional 1.18 billion people expected by 2050, for a total of 9.7 billion [1]. Anticipated protein needs are drawing concern, with forecasts indicating that global demand for protein from animal sources is set to double by the year 2050 [2], raising issues regarding sustainability and the assurance of food supply. This is partly due to the widespread understanding that foods derived from animals tend to emit more greenhouse gases (GHGs) compared to those from plants [3], which are linked to climate change. Moreover, the growing appetite for protein from animal sources is anticipated to escalate the strain on land resources to accommodate the expanded production of animal feed. Consequently, this expansion is likely to continue to contribute to the transformation of forests, wetlands, and natural grasslands into farmland. Such land conversion inherently carries adverse effects on GHG emissions, biodiversity, and various crucial ecosystem services [4]. From an environmental standpoint, plant-based proteins are favored over animal-based ones due to their association with reduced emission of GHG linked to climate change [3].

Soybean serves as an important source as plant-based protein [5] as well as animal feed. Soy accounts for over 60% of worldwide vegetable oil and protein consumption, and is the fourth-largest field crop in terms of volume [6]. Ongoing advancements in the genetic improvement of soybeans are essential to fulfill the global need for plant-based protein and oil, with per capita soybean consumption expected to rise by approximately 17% by 2029 [7]. Therefore, a sustained increase in soybean yield is crucial not only for farmers and animal producers but also for consumers and the overall sustainability of global agriculture. Accurately capturing phenotypic data is essential for the development of improved soybean genotypes, serving as a fundamental step toward increasing soybean yields.

Seed number per plant and weight per seed are the primary components determining grain yield of a soybean plant. Among these, seed number per unit of land area is overwhelmingly identified as the most critical factor influencing yield [8–10]. Therefore, developing automated tools for counting soybean seeds can greatly improve granular yield estimates before harvesting and may accelerate genetic improvement. Additionally, shoot architecture—the 3-dimensional structure of a plant’s above-ground organs—substantially impacts plant productivity in different planting densities, lodging risk, competition with weeds, and ultimately grain yield. These effects are related to light capture, photosynthetic efficiency, reactions to agronomic treatments, and adaptation to environmental conditions [11]. To a large degree, seed localization is contingent upon shoot architecture and is therefore not only a valuable selection criteria in itself but also a convenient proxy for shoot architecture. Quantifying seed localization is thus useful for multiple breeding objectives.

However, traditional approaches to analyzing shoot architecture and seed localization depend largely on manual counting and visual inspections—methods that are both labor-intensive and susceptible to human error and variability [12]. A more efficient method for estimating shoot architecture, estimating single plant yield and seed localization would significantly benefit breeders by providing novel, high-throughput phenotypes to help optimize shoot architecture and seed distribution to specific cropping systems.

Deep learning technologies have introduced novel pathways for automating these tasks, offering high-throughput, precise, and consistent analyses [13]. Utilizing these technologies in plant phenotyping can significantly boost the efficiency of breeding programs and aid in comprehending phenotypic response to environment. The common method in deep learning for object counting typically involves an initial step of object detection, followed by the counting of predicted bounding boxes [14]. However, this method requires labor-intensive and costly bounding box annotations. Furthermore, when objects are situated closely together, suppressing overlapping bounding boxes becomes a challenge [15]. Direct counting via regression represents an alternative technique within deep learning for object counting, which directly correlates counts to the target image using point labels as a guide [16,17]. Despite its efficiency, this approach falls short of revealing the precise locations of the counted objects, rendering the process somewhat opaque regarding both the nature and the localization of the counted objects within the image. The TasselNet series [18–20] aims to precisely count the number of tassels on corn plants employing regression-based techniques, yet the density maps produced for visualizing the predicted objects often lack intuitive explanation. The recent proposed P2PNet-Soy [17], which incorporates several advancements based on P2PNet [21], improved seed counting and localization by including unsupervised clustering to merge closely located overcounts and implementing channel and spatial attention mechanisms to effectively separate foreground and background. Despite these advancements, P2P-Soy still relies heavily on postprocessing techniques and exhibits significant overcounting issues, with its attention module—for separating foreground and background regions—demonstrating unstable performance on new data.

This study aims to bridge these gaps by incorporating a novel deep learning network architecture that improves both counting and localization accuracy of the network. The main contributions of this study are summarized as follows:

• We propose a multi-scale, attention mechanism-based deep learning framework, tailored to the precise counting and localization of soybean seeds on fully mature plants in the field.

• We test MSANet on 3 independent multi-season datasets that were not part of the training, showing the generalization ability of MSANet.

• We compared our model with the previous state-of-the-art model using not only on the benchmark dataset but also an enlarged dataset including various soybean genotypes.

• Our analysis further explores the phenotypic diversity among different soybean genotypes, providing novel insights into how these variations manifest in soybean seed characteristics.

## Materials and Methods

### Datasets

**Fig. 1.**
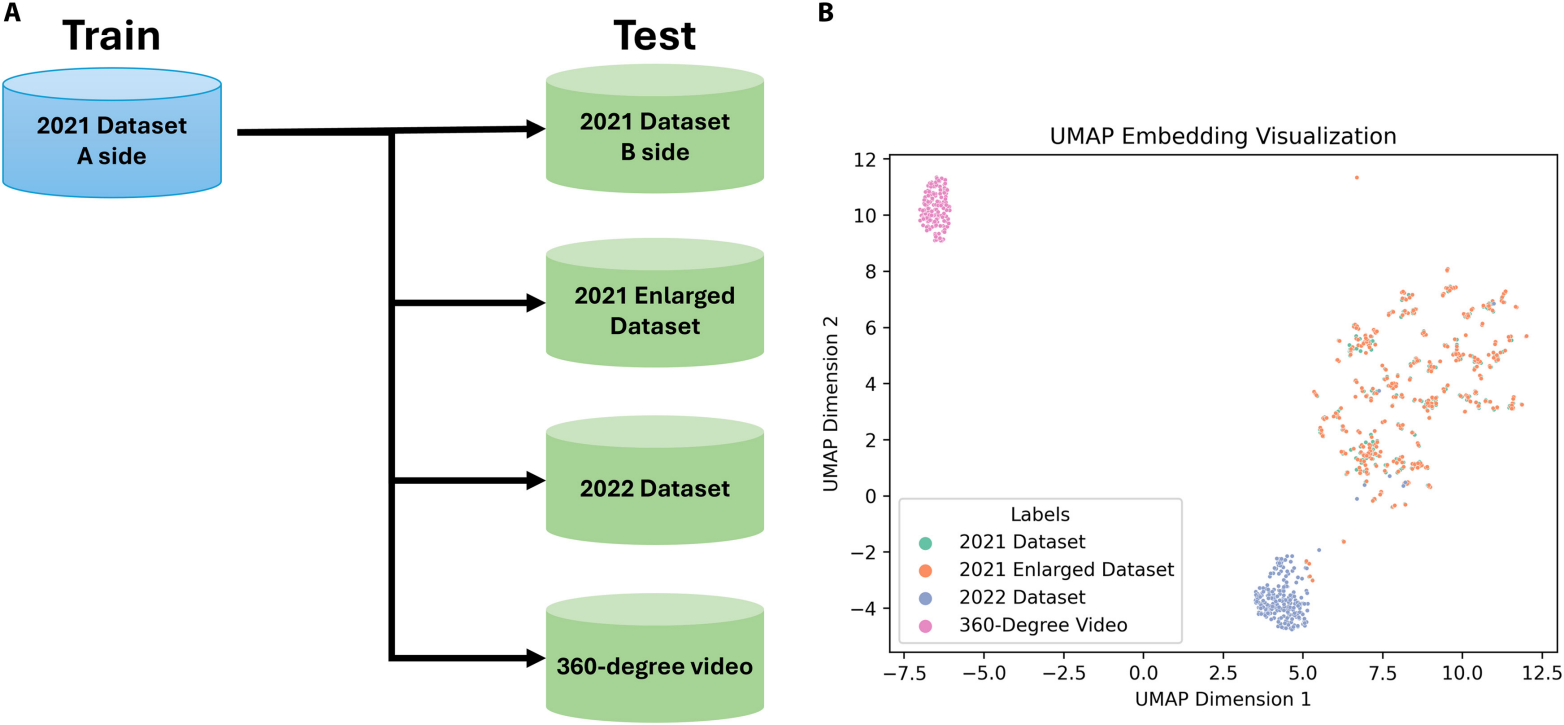
Overview of the datasets. (A) Overview of the roles of various datasets used in this study. The 2021 Dataset A side is used for training, while the 2021 Dataset B side, 2021 Enlarged Dataset, 2022 Dataset, and 360° video datasets are utilized for testing. (B) UMAP embedding visualization illustrating the dimensionality reduction and clustering of the different datasets.

The datasets used in this study play distinct roles in training and testing our models, as summarized in Fig. 1A. The 2021 Dataset A side is employed for training purposes, while the 2021 Dataset B side, the 2021 Enlarged Dataset, the 2022 Dataset, and the 360° video datasets are utilized for testing.

To gain insights into the relationships and characteristics of these datasets, we employed Uniform Manifold Approximation and Projection (UMAP) [22] for dimensionality reduction and visualization, as shown in Fig. 1B. From the UMAP visualization, it is evident that the 2021 Dataset and the 2021 Enlarged Dataset exhibit close proximity, indicating their similarity. The 2021 Enlarged Dataset, however, covers a broader range than the 2021 Dataset, effectively serving as an extended version of it. In contrast, the 2022 Dataset displays markedly different features, situated far from the 2021 Dataset in the UMAP plot, highlighting its distinct characteristics. The 360° video dataset is even more different, being much further away from both the 2021 and 2022 datasets, indicating that it is a completely different type of data. This clear separation highlights the unique nature of the 360° video data compared to the other datasets.

**Fig. 2.**
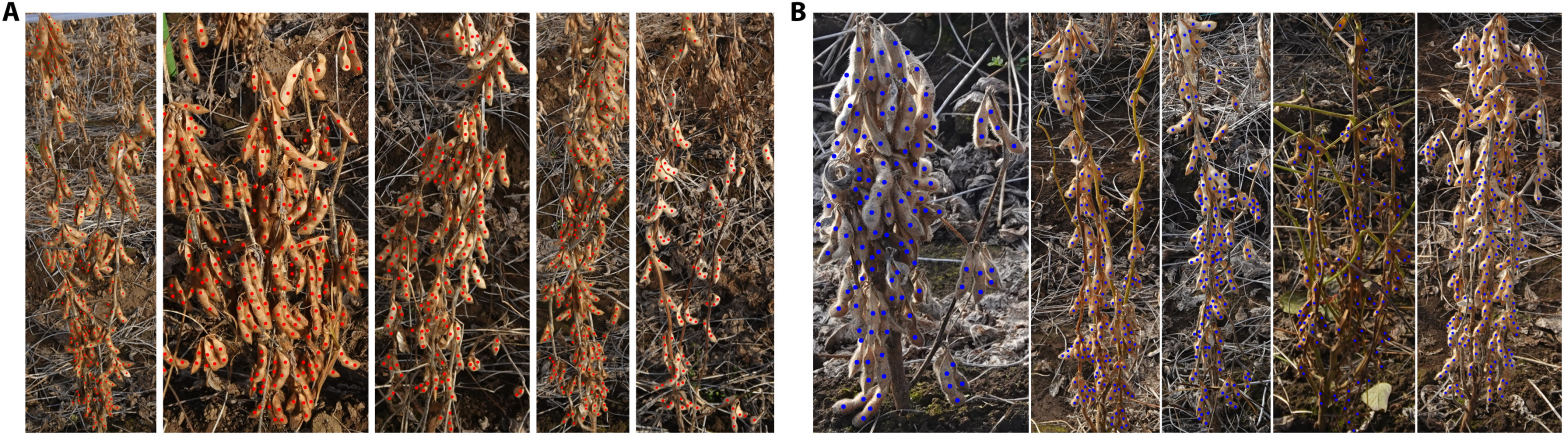
Samples of point-annotated cropped individual soybean plants from 2021 Dataset. (A) Annotated individual plants from one side. (B) Annotated individual plants from the opposite side.

Dataset 2021, which was used as the initial training and testing dataset, as originally used in Zhao et al.’s work [17], consists of a total of 258 images of individual soybean plants cropped from the plot images (Fig. 1), with point annotation of soybean seeds belonging to the target cropped individual plant, while seeds from the neighboring plants and background are ignored. As shown in Fig. 2, the same training set with cropped individual plant images taken from one side from 2021 Dataset (126 in total) were used, and 132 images from the opposite side were used for evaluation.

**Fig. 3.**
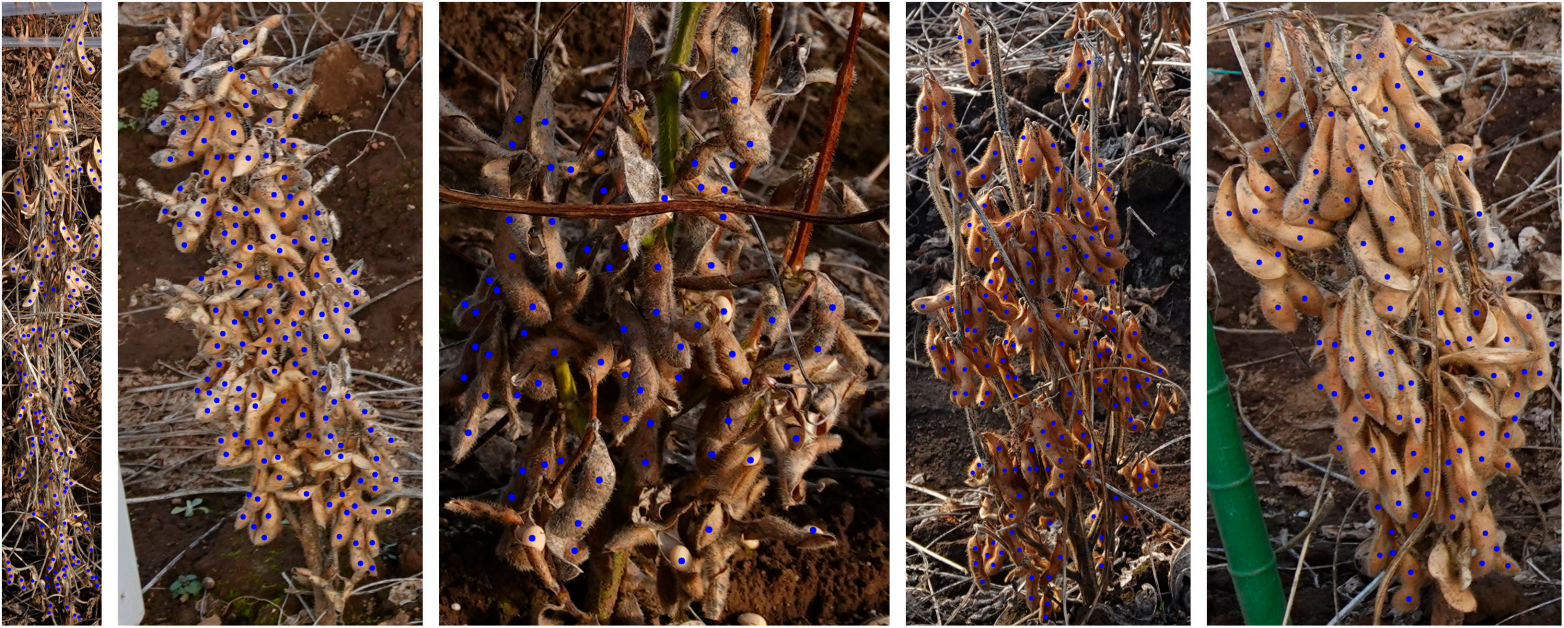
Samples of point-annotated cropped individual soybean plants from 2021 Enlarged Dataset.

Complementing this, 2021 Enlarged Dataset as shown in Fig. 3 offers a broader collection of 378 images. This dataset was gathered using the same methodology and during the same season and location as Dataset 2021, as detailed in [17]. It aims to provide a more extensive perspective on the plants while maintaining consistency with the field conditions and experimental design described in [17].

**Fig. 4.**
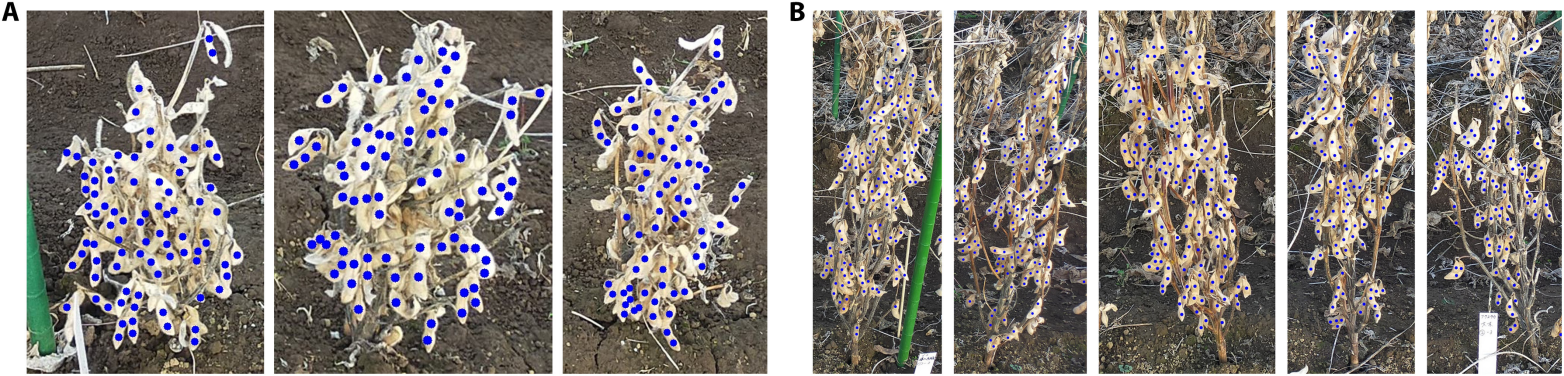
Samples of point-annotated cropped individual soybean plants from 2022 Dataset. (A) Annotated individual plants from genotype *N24E*. (B) Annotated individual plants from genotype *M307*.

The 2022 Dataset contains 250 images of individual soybean plants collected from a field at National Agriculture and Food Research Organization (NARO), Tsukuba, Ibaraki (Japan) and follows the same data collection pipeline as in 2021 Dataset. A total of 25 genotypes were selected in this study (12 accessions that are logging or share severe overlaps with individual plants were excluded in this experiment). Figure 4 shows samples from genotype *N24E* and *M307*. In-field images from both sides of the 5 plants per plot were captured at full physiological maturity using a handheld camera (DSC-RX0M2, Sony, Japan) from a shooting position approximately 30 cm horizontally and 150 cm vertically from the target row. Individual soybean plant images were then cropped manually with Labelme [23], and the seeds of each plant were point-annotated as shown in Fig. 4 by experienced technicians from the Fieldphenomics Lab at the University of Tokyo. We applied the same preprocessing strategy as Zhao et al. [17], which involves only annotating seeds that belong to the targeted cropped individual plant and ignoring seeds from the neighboring plants and the background.

The 360° video dataset, collected in 2023, is another critical component of our testing datasets. These data waere gathered at the NARO in Tsukuba, Ibaraki, Japan, the same location as the 2022 Dataset. Using a cart equipped with an Insta360 X3, a cutting-edge 360° camera, we captured video footage of multiple rows in a soybean breeding field, which included a diverse set of germplasm. For the purpose of this study, we randomly selected one row from the captured footage for testing. This innovative data collection method not only allowed us to test the model’s performance on entirely unseen data but also significantly improved data collection efficiency. The 360° video dataset presents markedly different features from the other datasets, as evidenced by its distinct positioning in the UMAP visualization (Fig. 1B). This clear separation highlights the unique nature of the 360° video data compared to the other datasets, providing a robust test for the generalizability and versatility of our model.

### Network design

Our proposed multi-scale attention network (MSANet) addresses the double-counting issue and enhances accuracy in both counting and locating soybean seeds. MSANet works in 2 parts: firstly, it generates heatmaps that illustrate seed distribution; secondly, it employs a multi-scale attention mechanism. By creating seed distribution heatmaps, MSANet converts the task of soybean seed counting and localization from a key point detection task to a segmentation task. Furthermore, the multi-scale attention mechanism directs the neural network to prioritize the foreground by using attention maps, which helps to distinguish seeds more effectively from the complex background.

#### Creation of seed distribution heatmaps

In our soybean seed detection task, we begin by transforming sparse point annotations, representing the centroids of individual soybean seeds, into binary images of the original image dimensions. Each annotated point is converted into a pixel with a value of 1 within these binary images, thus creating precise markers for the presence of soybean seeds.

Let **P**  = {(*x_i_*,  *y_i_*)} represent the set of coordinate points in the image. For each point (*x_i_*,  *y_i_*) in **P**, morphological dilation is first applied to the keypoint heatmap to generate the dilated heatmap *H_d_*, which can be represented as:

$$H_{d}\left(x,y\right)=\underset{\left(a,b\right)\in B}{\text{max}}H\left(x+a,\;y+b\right)$$ (1)

where *H*(*x*, *y*) is the initial heatmap representation of the points in **P**, and **B** is the structuring element used for dilation.

**Fig. 5.**
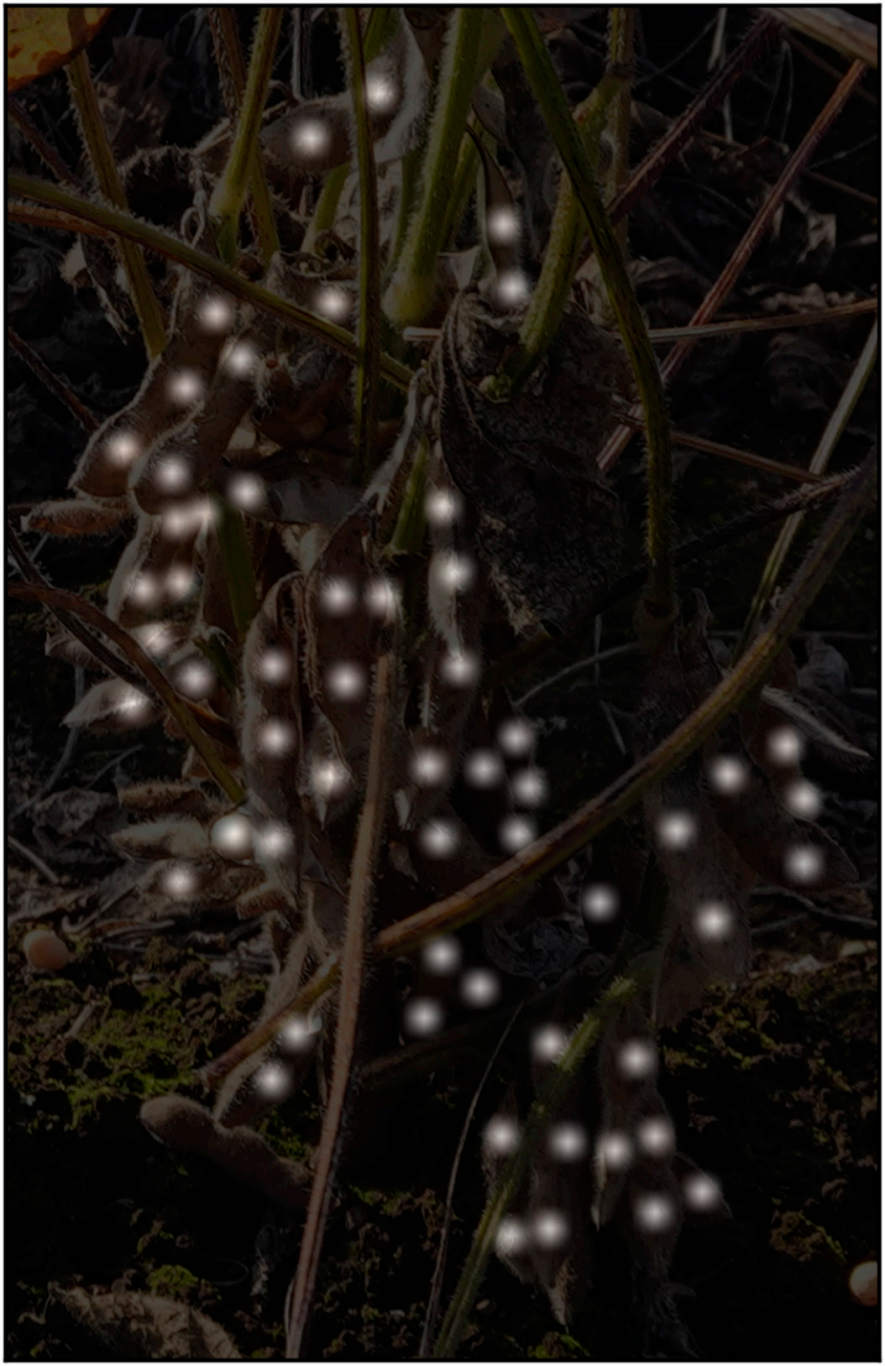
Visualization of heatmap annotation.

Following the initial transformation, we employ morphological dilation on the binary images to emphasize the spatial location of each seed. This process is succeeded by the application of Gaussian filtering, which diffuses the binary markers into continuous heatmaps, depicting the likelihood of seed presence with the points close to the center being 1.0 and points farther from the center ranging gradually becoming 0.0. Figure 5 shows an example of heatmap annotation. This approach elegantly captures the spatial distribution of soybean seeds, providing a detailed probability landscape for the subsequent stages of our model.

Then, Gaussian smoothing is then applied to the dilated heatmap to generate the smoothed heatmap *H_s_*:

$$H_{s}=H_{d}*G$$ (2)

where *G*(*x*, *y*) is Gaussian filter defined as:

$$G\left(x,y\right)=\frac{1}{2{\pi \sigma }^{2}}\text{exp}\left(-\frac{x^{2}+y^{2}}{2\sigma^{2}}\right)$$ (3)

where *σ* is the standard deviation of the Gaussian filter.

#### Multi-scale deep learning framework design

**Fig. 6.**
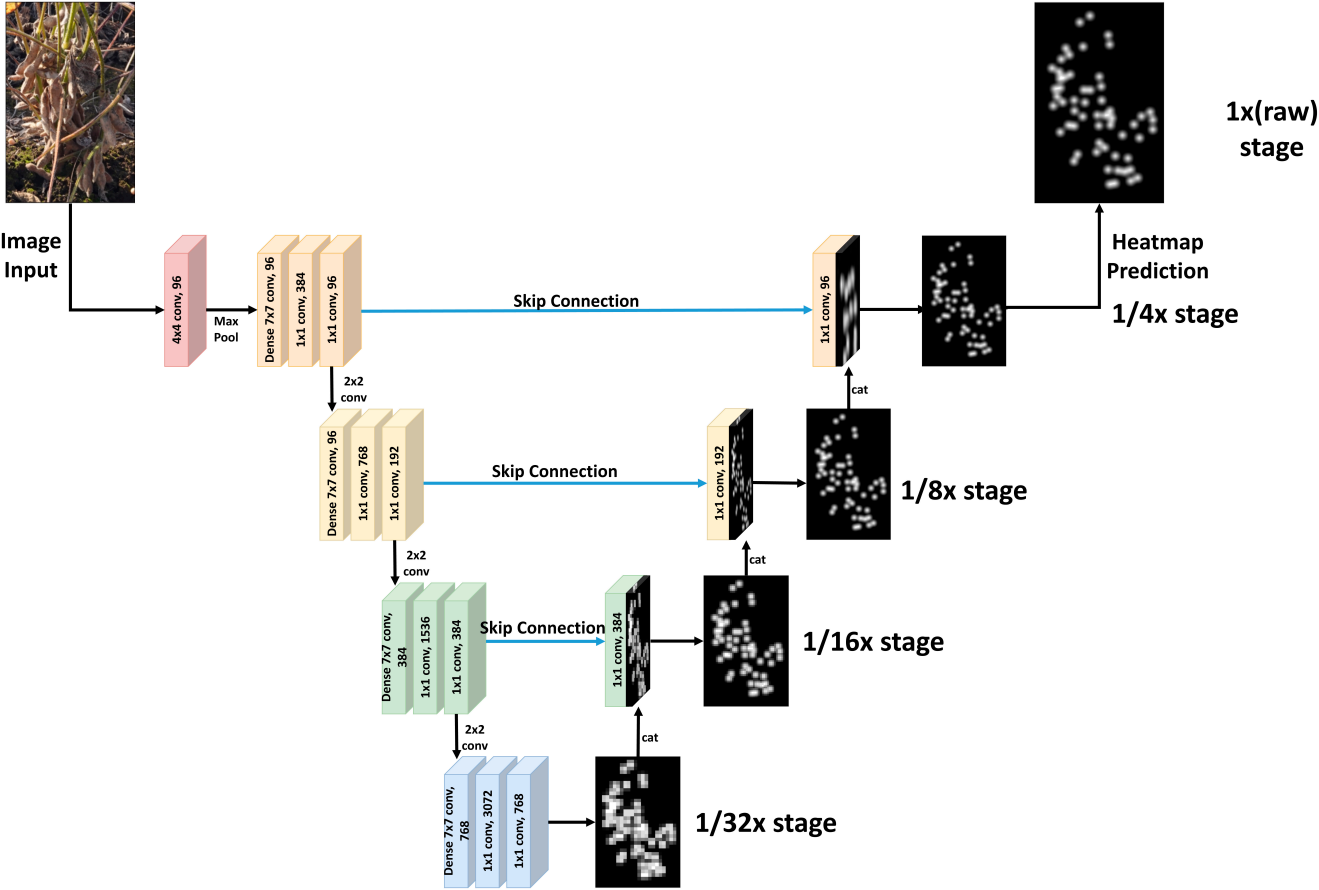
The overall architecture of the proposed model. Built upon the Convnext as the backbone network, the first step is firstly to introduce a downsampling path to obtain fine-gained deep feature map across 4 stages, yielding multi-scale representations. Each stage generates a predictive output, which is then upsampled and reintegrated to the original resolution through skip connections. These connections enhance the feature maps by concatenating intermediate stage predictions, facilitating the synthesis of the final output.

As illustrated in Fig. 6, our proposed model leverages an encoder–decoder structure, inspired by various works in semantic segmentation and medical image analysis [24–26], which has proven effective in preserving spatial information and enabling detailed reconstructions. Specifically, our model introduces a multi-scale attention mechanism that enhances the network’s ability to focus on relevant features at different resolutions, thus improving its performance in distinguishing soybean seeds from the background. This multi-scale attention framework, integrated within the encoder–decoder structure, ensures that the network remains attentive to significant features across varying scales, providing a robust and precise detection capability.

We use the convnext_small [27,28], excluding the classification head to extract deep features for different scales S = 1/4, 1/8, 1/16, 1/32, which methodically downsamples the image across 4 distinct stages to create a series of progressively reduced scales. It is worth noting that convnext is pretrained on ImageNet and then fine-tuned on our dataset. With the output feature map, we upsampled its spatial resolution by a factor of 2 using nearest neighbor interpolation. Each stage autonomously generates a predictive output, which is subsequently upsampled and merged back to match the original image scale. Upsampling in this way utilizes skip connections to group cluster predictions from each stage with the feature map, which aids in refining the final result.

Real-time training with a batch size of one was implemented during the training phase in order to further harness the potential of this multi-scale attention mechanism on a technical level. This approach allows the model to process images in their original resolution without the need for resizing or cropping, preserving the integrity and full scope of the soybean plant imagery. This strategy enables the model to effectively combine detailed features with broader contextual information, a fusion critical for accurately performing tasks that require close attention to detail, such as detailed counting in an agricultural context.

#### Attention maps naturally emerge using a foreground focus technique

**Fig. 7.**
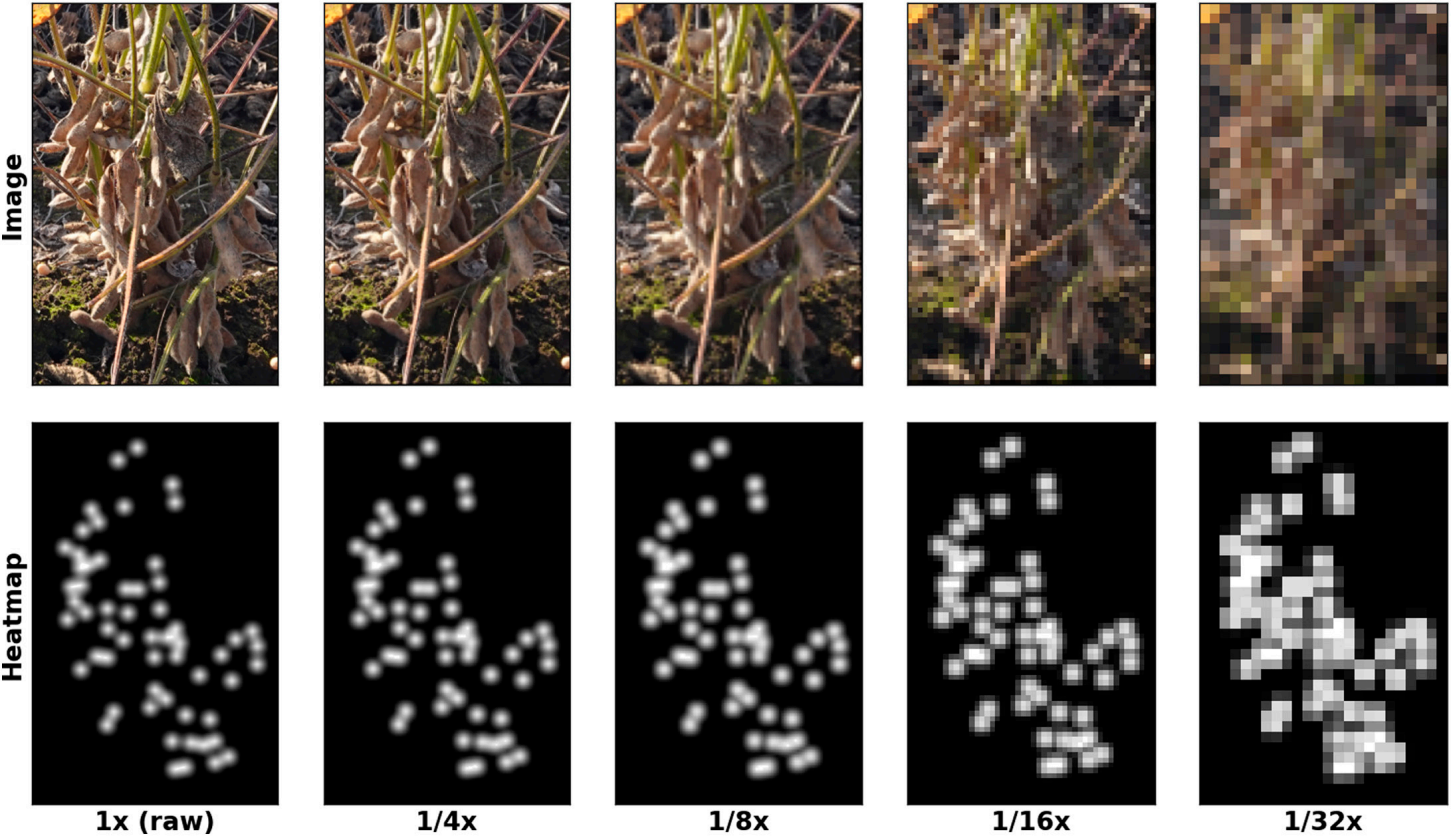
Visualization of downsampled image and heatmap. The top row displays the original soybean image progressively downsampled to lower resolutions: 1× (input), 1/4×, 1/8×, 1/16×, and 1/32×. The bottom row presents the corresponding heatmaps, where the brightness indicates the density of soybean seeds.

Aligning with the multi-scale approach of our network, the generated heatmaps are then downsampled to match the resolutions of 5 distinct feature maps produced by the network (Fig. 7). This ensures that our model maintains a consistent representation of seed distribution across varying levels of detail. The multi-scale feature maps enable the network to capture both fine-grained and coarse-grained details, allowing it to accurately detect soybean seeds at different scales.

The unique aspect of our approach lies in the natural formation of attention-like maps as a result of downsampling the high-resolution probability maps. This multi-scale attention mechanism inherently guides the network to focus on the most relevant areas across different resolutions. Since only the foreground soybean seeds were annotated, disregarding the background, the downsampled maps at lower resolutions (as shown in Fig. 7, 1/32×) inherently resemble attention maps that highlight the entire foreground soybean plants. This resemblance is not coincidental but a deliberate design choice that encourages the network to concentrate on the foreground entities, thereby enhancing the detection accuracy of individual soybean seeds against the complex backdrop of overlapping plants. The multi-scale attention framework ensures that the network remains attentive to significant features at each scale, improving the overall robustness and precision of seed detection.

#### Loss function

The loss function design for our proposed model utilizes a multi-scale approach to compute the mean square error (MSE) at different scales. The model output result predictions correspond to feature maps at varying scales. These are compared against the ground-truth labels, which are downsampled to the respective scales using max pooling. The MSE is calculated for each scale between the predicted output and the corresponding downsampled labels. The total training loss is the sum of the individual losses across all scales, ensuring that the model’s predictions are accurate at multiple levels of granularity.

Let *GT* and *P* represent the ground truth and predicted outputs at the original image scale, while *GT^s^* and *P^s^* represent the ground truth and prediction at scale *s* within the set of scale *S*.

For the semantic segmentation loss at a single scale, it is defined as:

$$L_{\mathrm{seg}}=\mathrm{MSE}\left(\mathrm{GT},\;P\right)=\frac{1}{N}\sum_{i=1}^{N}{\left({\mathrm{GT}}_{i}-P_{i}\right)}^{2}$$ (4)

where *i* represents the *i*th pixel in the ground-truth segmentation map.

For the multi-scale loss, which accounts for the errors across all scales, it is defined as:

$$L_{\mathrm{ms}}=\sum_{s\in S}\left(\mathrm{MSE}\left({\mathrm{GT}}^{s}-P^{s}\right)\right)$$ (5)

where *i* represents the *i*th pixel in the ground-truth segmentation map.

The total loss, *L*, is then the sum of the segmentation and multi-scale losses:

$$L=L_{\mathrm{seg}}+\lambda L_{\mathrm{ms}}$$ (6)

where *λ* is a weight term to balance the effect of 2 losses. In our case, the value of *λ* was chosen as 1 to equalize the contributions of both loss components.

### Evaluation metrics

Our model performance was evaluated through a 2-pronged approach, addressing both counting and localization tasks. This dual-evaluation strategy provided a holistic assessment of the model’s capabilities in detecting and accurately positioning soybean seeds within field images. Each task was assessed using distinct, task-specific metrics, ensuring a detailed and targeted evaluation of the model’s performance.

#### Counting task evaluation

For the counting task, we focused on the model’s ability to estimate the correct number of seeds. Three key metrics were used for this evaluation:

• Coefficient of determination (*R*^2^): This metric provides a measure of how well the observed outcomes are replicated by the model, based on the proportion of total variation of outcomes explained by the model.

• Mean absolute error (MAE): MAE gives a straightforward average of absolute differences between predicted and actual counts, serving as a clear indicator of counting accuracy.

• Root mean square error (RMSE): RMSE calculates the average root of the squares of the errors, which penalizes larger errors more severely and provides insight into the variance of the count predictions.

#### Localization task evaluation

The evaluation of the localization task delved into the precision of the model’s ability to determine seed locations. Each of the metrics defined above provide insights into different aspects of the model’s localization performance, from the average accuracy of placement to the ability to detect and correctly identify the presence of seeds.

• Mean Euclidean distance (MED): MED is computed by matching predicted coordinates with ground-truth coordinates using the nearest neighbor method. Once pairs are established, we calculate the MED between each pair, with smaller values indicating more precise localizations.

• Precision: Precision is calculated as the ratio of the number of correctly matched pairs to the total number of predicted coordinates. This metric reflects the proportion of predicted coordinates that were accurate, highlighting the model’s specificity.

• Recall: Recall is determined by the ratio of the number of correctly matched pairs to the total number of ground-truth coordinates. It indicates the model’s sensitivity by showing what proportion of actual seed locations were correctly identified and localized by the model.

• F1 score: The F1 score is a combined measure of precision and recall, offering a balanced view of a model’s accuracy. It is particularly valuable when minimizing both false positives (FPs) and false negatives (FNs) is crucial. This metric simplifies the evaluation of a model’s overall performance, facilitating easier comparison between models.

#### Matching algorithm for localization accuracy

To accurately compute the localization metrics of MED, precision, and recall, a prerequisite step involves the matching of predicted coordinates to ground-truth coordinates. This process enables us to determine true positives (TPs), FPs, and FNs crucial for these calculations.

The matching algorithm employs a nearest-neighbor analysis to determine correspondences between detected points, considering a match when the pixel distance is within the empirically determined threshold. We chose 25 pixels as the threshold maximum for matching, because this corresponded well with the average distance among neighboring seeds in real-world soybean conditions.


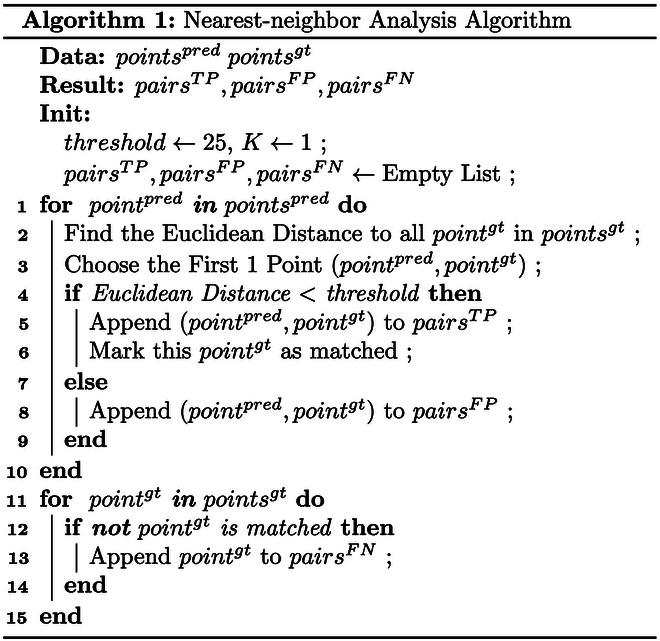


Let **G** = {(*x_i_*, *y_i_*)} represent the set of ground-truth coordinate points in the image and $P=\left\{\left({\hat{x}}_{i},\;{\hat{y}}_{i}\right)\right\}$ represent the set of predicted coordinate points in the image. For each image, MED can be calculated as:

$$\mathrm{MED}=\frac{1}{M}\sum_{i=1}^{M}\sqrt{{\left(x_{i}-{\hat{x}}_{i}\right)}^{2}+{\left(y_{i}-{\hat{y}}_{i}\right)}^{2}}$$ (7)

where *M* is the number of matched point pairs and *i* represents the *i*th pixel in the image. Then, for all images in the dataset:

$$\bar{\mathrm{MED}}=\frac{1}{N}\sum_{i=1}^{N}{\mathrm{MED}}^{i}$$ (8)

where *N* is the number of images in the dataset and *i* represents the *i*th image in the dataset.

Precision and recall can be calculated as follows:

$$\text{Precision}=\frac{\mathrm{TP}}{\mathrm{TP}+\mathrm{FP}}$$ (9)

$$\text{Recall}=\frac{\mathrm{TP}}{\mathrm{TP}+\mathrm{FN}}$$ (10)

$$F1\;\text{score}=\frac{2\cdot \text{Precision}\cdot \text{Recall}}{\text{Precision}+\text{Recall}}$$ (11)

#### Kernel density estimate

In this paper, we propose new trait focuses on the vertical seed distribution of soybean plant, a single trait derived from the kernel density estimate (KDE) curves of seeds’ longitudinal (*y*-axis) positions.

KDE is a nonparametric method used to estimate the probability density function of a random variable [29]. The method has been formalized in foundational works by Rosenblatt [30] and Parzen [31], which is especially useful for visualizing the distribution of data points in a smooth manner, without assuming any underlying distribution. KDE works by placing a kernel (which is a smooth, bell-shaped curve) on each data point and then averaging all these kernels to produce a density estimate. The result is a smooth curve that represents the data’s distribution.

$$f\left(x\right)=\frac{1}{n}\sum_{i=1}^{n}\frac{1}{h}K\left(\frac{x-x_{i}}{h}\right)$$ (12)

where *f*(*x*) is the estimated density at point *x*, (*n*) is the number of data points, *x_i_* are the data points, *h* is the bandwidth, which determines the width of the kernel and thus controls the smoothness of the resulting density curve, and *K*(⋅) represents the Gaussian kernel function. In our analysis, we employed Scott’s rule [32] to automatically determine the bandwidth, which is a widely used method for bandwidth selection in KDE. Scott’s rule adjusts the bandwidth proportionally to *n*^−1/5^, aiming to minimize the mean integrated squared error (MISE) of the density estimate. This approach balances the trade-off between the bias and variance of the estimate, providing a practical and theoretically grounded method for selecting an appropriate bandwidth.

To quantify the distance between the KDE of ground truth and the predictions made by MSANet, we use the Kullback–Leibler (KL) divergence, which is a measure from information theory and quantifies how one probability distribution diverges from a second reference probability distribution.

The KL divergence is defined as:

$$D_{\mathrm{KL}}\left(P\|Q\right)=\int_{-\infty }^{\infty }p\left(x\right)\text{log}\left(\frac{p\left(x\right)}{q\left(x\right)}\right)\mathrm{dx}$$ (13)

where *P* and *Q* represent the ground truth and predicted distributions, respectively, with *p*(*x*) and *q*(*x*) their corresponding density functions. This definition emphasizes the asymmetry of KL divergence: *D_KL_*(*P*‖*Q*) ≠ *D_KL_*(*Q*‖*P*), which highlights its nature as a “distance” from *P* to *Q*, in terms of information content.

### Ablation study

In addition to studying the overall performance of our proposed model, it is crucial to understand how different stages of the MSANet contribute to the final results. To analyze the impact of each stage, we conducted an ablation study, where we systematically blocked different stages of the MSANet, starting from the smallest 1/32 stage and gradually blocking up to the 1/4 stage, ultimately using only the raw stage. This method allowed us to isolate the effect of each stage and determine its significance in contributing to the overall accuracy and robustness of the model. By evaluating the performance variations with each stage blocked, we aimed to identify which stages were most critical to the model’s effectiveness in detecting and distinguishing soybean seeds from the background.

## Results

In this work, we conducted a thorough evaluation of MSANet on the 2021 Dataset, the 2021 Enlarged Dataset, and the 2022 Dataset, utilizing the evaluation metrics introduced in the “Evaluation metrics” section.

We compared the MSANet against the previously proposed state-of-the-art model, P2PNet-Soy [17], designed specifically for soybean seed counting and localization based on the P2PNet [21] framework by incorporating several innovations. It addresses overcounting through KD-Tree postprocessing and enhances feature representation by integrating input imagery with channel and spatial attention. Given the specific design and optimization of P2PNet-Soy for soybean seed counting, we consider it one of the most relevant comparative methods.

Furthermore, recognizing that MSANet is built around an optimized multi-scale attention mechanism and outputs a heatmap, we additionally perform comparisons with the DeepLabv3+ model [33], a well-established semantic segmentation model by replacing the heatmap extraction part of MSANet with it. This allowed us to validate the reliability and robustness of the multi-scale attention structure in a different context.

### 2021 Dataset evaluation

**Fig. 8.**
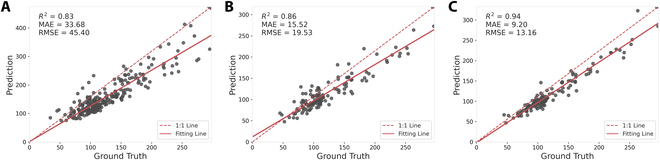
Performance comparison between DeepLabv3+, P2PNet-Soy, and MSANet on the 2021 Dataset. (A) DeepLabv3+. (B) P2PNet-Soy. (C) MSANet.

In the evaluation of our models for soybean seed detection, the MSANet model demonstrated superior performance across most metrics compared to both the P2PNet-Soy [17] model and the DeepLabv3+ [33] model, as shown in Table 1. Figure 8 visualizes the performance comparison between P2PNet-Soy and MSANet on the soybean seed counting task.

**Table 1.** Evaluation on the 2021 Dataset. Note that the arrow upward indicates the higher, the better, whereas the arrow downward indicates the lower, the better.

	Counts			Localization			
	*R*^2^↑	MAE↓	RMSE↓	$\bar{\mathrm{MED}}↓$	Precision↑	Recall↑	F1 score↑
DeepLabv3+ [33]	0.83	33.68	45.40	6.75	0.73	0.90	0.81
P2PNet-Soy [17]	0.86	15.52	19.53	8.08	0.79	0.75	0.77
MSANet	0.94	9.20	13.16	7.52	0.87	0.85	0.86

Specifically, for the counting task evaluation, MSANet achieved a higher *R*^2^ value of 0.94, indicating a stronger correlation between the predicted and actual data, compared to 0.86 for P2PNet-Soy and 0.83 for DeepLabv3+, respectively. Moreover, MSANet exhibited greater accuracy with a lower MAE of 9.20 and RMSE of 13.16, in contrast to the P2PNet-Soy, which had an MAE of 15.52 and an RMSE of 19.53, and DeepLabv3+, which had an MAE of 33.68 and an RMSE of 45.40.

For the localization task evaluation, MSANet’s MED metric was lower at 7.52, suggesting a tighter error distribution than both P2PNet-Soy, which had a MED of 8.08, and DeepLabv3+, which had a MED of 6.75. Importantly, MSANet outperformed in precision with a score of 0.87 and recall with 0.85, leading to a higher F1 score of 0.86 for localizing the soybean seeds, compared to the F1 scores of 0.77 for P2PNet-Soy and 0.81 for DeepLabv3+.

These results underscore the effectiveness of MSANet in the precise and sensitive identification of soybean seed locations in the given dataset.

### 2021 Enlarged Dataset evaluation

**Fig. 9.**
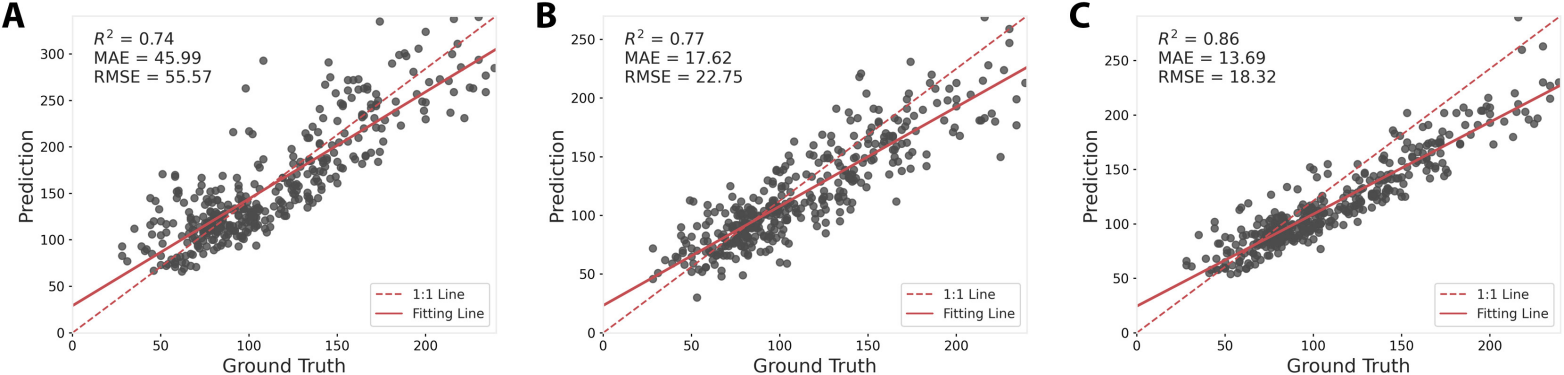
Performance comparison between DeepLabv3+, P2PNet-Soy, and MSANet on the 2021 Enlarged Dataset. (A) DeepLabv3+. (B) P2PNet-Soy. (C) MSANet.

For the 2021 Enlarged Dataset, the evaluation of our model also revealed a clear distinction in performance across both counting and localization tasks as shown in Table 2. Figure 9 visualizes the performance comparison between P2PNet-Soy and MSANet on the soybean seed counting task.

**Table 2.** Evaluation on the 2021 Enlarged Dataset. Note that the arrow upward indicates the higher, the better, whereas the arrow downward indicates the lower, the better.

	Counts			Localization			
	*R*^2^↑	MAE↓	RMSE↓	$\bar{\mathrm{MED}}↓$	Precision↑	Recall↑	F1 score↑
DeepLabv3+ [33]	0.74	45.99	55.57	7.75	0.64	0.89	0.74
P2PNet-Soy [17]	0.77	17.62	22.75	8.25	0.76	0.80	0.78
MSANet	0.86	13.69	18.32	8.08	0.81	0.87	0.84

For the counting task evaluation, the MSANet model significantly improved over the P2PNet-Soy and DeepLabv3+ models. MSANet achieved a higher *R*^2^ value of 0.86, suggesting a stronger correlation with the actual data points compared to 0.77 by the P2PNet-Soy and 0.74 by DeepLabv3+, respectively. The MAE and RMSE for MSANet were 13.69 and 18.32, respectively, indicating a higher prediction accuracy and consistency than the P2PNet-Soy model, which reported an MAE of 17.62 and an RMSE of 22.75, and DeepLabv3+, which had an MAE of 45.99 and an RMSE of 55.57.

Moving to the localization task evaluation, MSANet again outperformed both P2PNet-Soy and DeepLabv3+. The MED for MSANet was 8.08, slightly lower than the 8.25 from P2PNet-Soy, but higher than the 7.75 from DeepLabv3+, indicating a generally accurate localization of the soybean seeds. Precision and recall are crucial for determining the model’s effectiveness in localization; MSANet achieved 0.81 and 0.87, respectively, surpassing both P2PNet-Soy, which had scores of 0.76 and 0.80, and DeepLabv3+, which had scores of 0.64 and 0.89. This resulted in a higher F1 score of 0.84 for MSANet, compared to 0.78 for P2PNet-Soy and 0.74 for DeepLabv3+, illustrating its better balance between precision and recall.

### 2022 Dataset evaluation

**Fig. 10.**
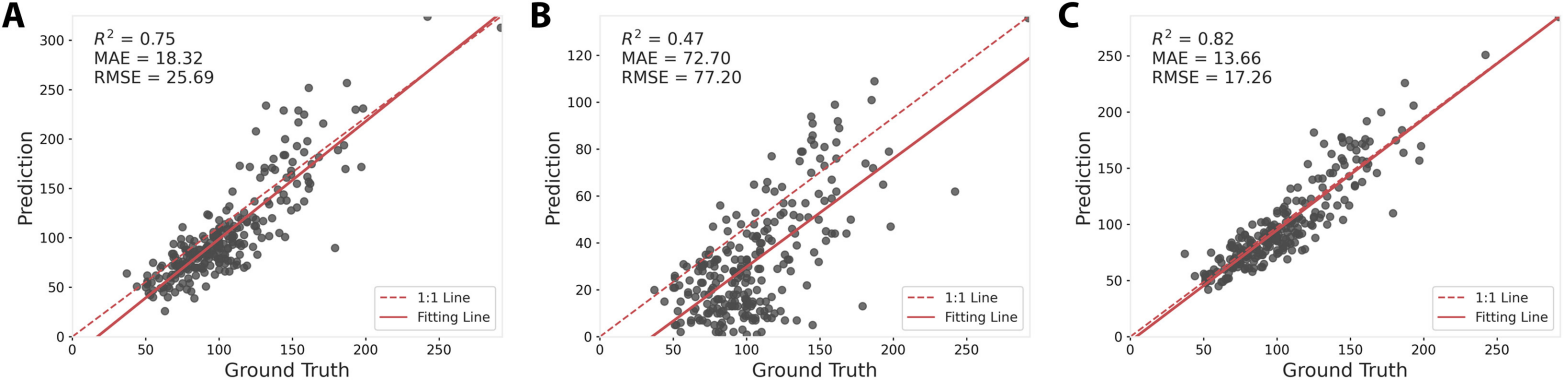
Performance comparison between DeepLabv3+, P2PNet-Soy, and MSANet on the 2022 Dataset. (A) DeepLabv3+. (B) P2PNet-Soy. (C) MSANet.

In the 2022 Dataset evaluation, for the counting task, the MSANet model demonstrated an *R*^2^ value of 0.82, with an MAE of 13.66 and an RMSE of 17.26, outperforming both the P2PNet-Soy and DeepLabv3+ models. The P2PNet-Soy model showed a significantly lower *R*^2^ of 0.47, with an MAE of 72.70 and an RMSE of 77.70, while DeepLabv3+ achieved an *R*^2^ of 0.75, with an MAE of 18.32 and an RMSE of 25.69, as shown in Table 3. Figure 10 visualizes the performance comparison between P2PNet-Soy and MSANet on the soybean seed counting task.

**Table 3.** Evaluation on the 2022 Dataset. Note that the arrow upward indicates the higher, the better, whereas the arrow downward indicates the lower, the better.

	Counts			Localization			
	*R*^2^↑	MAE↓	RMSE↓	$\bar{\mathrm{MED}}↓$	Precision↑	Recall↑	F1 score↑
DeepLabv3+ [33]	0.75	18.32	25.69	4.87	0.83	0.80	0.82
P2PNet-Soy [17]	0.47	72.70	77.20	4.87	0.96	0.28	0.43
MSANet	0.82	13.66	17.26	4.99	0.91	0.85	0.88

Evaluating the localization task across the 2022 Dataset, MSANet outperformed both P2PNet-Soy and DeepLabv3+ overall. MSANet achieved a precision of 0.91, a recall of 0.85, and an F1 score of 0.88. Although P2PNet-Soy had a higher precision of 0.96, its recall was only 0.28, resulting in a much lower F1 score of 0.43. DeepLabv3+ had a precision of 0.83, recall of 0.80, and an F1 score of 0.82. These results suggest that MSANet has a better balance between precision and recall, even on the new dataset, making it more effective overall for localization tasks.

The 2022 Dataset evaluation demonstrates the robustness of MSANet in both counting and localization tasks, with particularly notable performance in accurately detecting and localizing soybean seeds. These findings highlight the effectiveness of MSANet as a reliable model for the tasks of soybean seed detection and localization, regardless of generalization challenges presented by different genotype characteristics.

### 360° video

However, the performance of MSANet is largely influenced by the efficiency of data collection. Traditional cameras present several limitations that can hamper the data collection process. For instance, to capture the entirety of a plant, images had to be taken from the space between rows, which required maintaining a set distance. When adjacent row plants were large, it necessitated positioning the camera between these plants for shooting, making continuous shooting challenging, and in the case of video, individuals from the adjacent rows were also captured. Furthermore, capturing smaller plants required crouching to shoot from the side, necessitating adjustments in shooting angles for each individual, resulting in varying heights of capture. These variations could potentially influence the evaluation.

So, MSANet was also applied to video footage captured by a 360° camera to improve the data collection efficiency and to further demonstrate the versatility and potential of our approach. Utilizing a cart equipped with an Insta360 X3, a cutting-edge 360° camera, video of multiple rows in a soybean breeding field including a diverse set of germplasm was captured. This innovative method allowed us to test the model’s performance on entirely unseen data by applying it to the video footage for seed detection and also increased data collection efficiency dramatically.

**Fig. 11.**
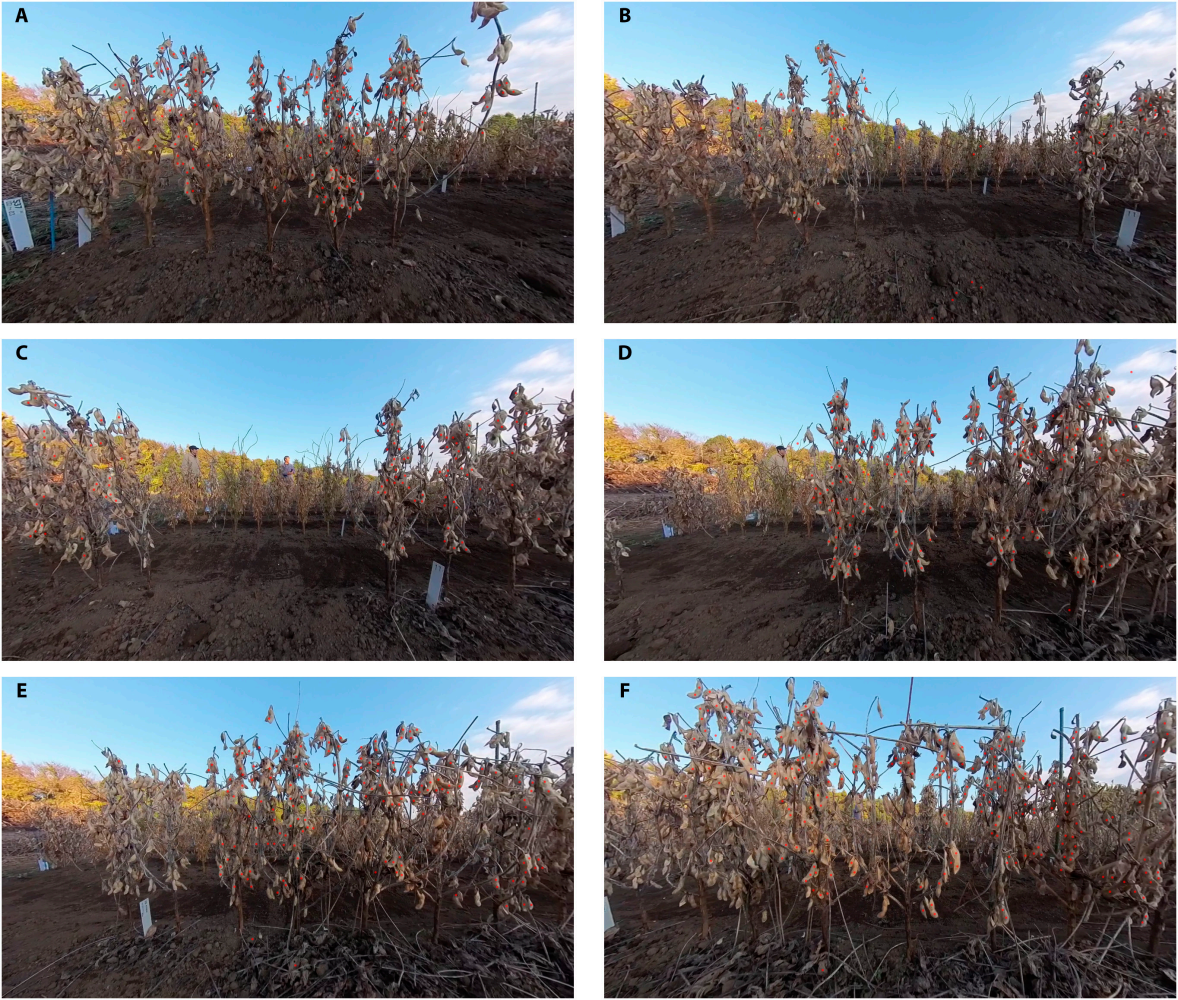
MSANet’s application to 360°, in canopy video. (A) to (F) are 6 frames progressively selected with a 100-frame interval from the video.

As shown in Fig. 11, the results indicate that, while the MSANet proficiently identified soybean seeds, it occasionally misclassified soil as soybean seeds. For the complete video, please refer to https://youtu.be/0y6zQuwkOT4.

Table 4 also provides the statistical comparison evaluation on the 360° video data between MSANet, DeepLabv3+, and P2PNet-Soy [17]. These results are based on 4 manually annotated frames from the video, providing a rough indication of the model’s performance. It is worth noting that during the annotation process, it was observed that the 360° video data present large differences compared to the previous datasets. The resolution is considerably lower, and due to slight motion blur, many soybean seeds are difficult to distinguish even with the human eye. These factors likely contributed to the overall performance metrics.

**Table 4.** Evaluation on the 360° video. Note that the arrow upward indicates the higher, the better, whereas the arrow downward indicates the lower, the better.

	Counts			Localization			
	*R*^2^↑	MAE↓	RMSE↓	$\bar{\mathrm{MED}}↓$	Precision↑	Recall↑	F1 score↑
DeepLabv3+ [33]	0.14	297.50	325.58	6.10	0.79	0.17	0.29
P2PNet-Soy [17]	0.32	361.00	387.69	11.20	0.06	0.00	0.01
MSANet	0.41	250.00	276.26	6.25	0.85	0.30	0.45

Despite these challenges, MSANet demonstrates a notable detection capability even with this different dataset. The *R*^2^ value for MSANet is 0.41, which is higher than both P2PNet-Soy’s 0.32 and DeepLabv3+’s 0.14, and it shows lower error metrics (MAE of 250.00 and RMSE of 276.26) compared to both P2PNet-Soy (MAE of 361.00 and RMSE of 387.69) and DeepLabv3+ (MAE of 297.50 and RMSE of 325.58). This indicates a better overall performance in counting metrics.

When examining localization metrics, the differences become even more pronounced. MSANet achieves a precision of 0.85 and a recall of 0.30, whereas P2PNet-Soy has a precision of 0.06 and a recall of 0.00, and DeepLabv3+ achieves a precision of 0.79 and a recall of 0.17. This highlights that MSANet can correctly identify a substantial number of soybean seeds, while P2PNet-Soy fails to detect them reliably and DeepLabv3+ struggles with recall. The F1 score further reflects this difference, with MSANet achieving 0.45 compared to P2PNet-Soy’s 0.01 and DeepLabv3+’s 0.29.

Although the median localization error (MED) for DeepLabv3+ is the lowest at 6.10, it is slightly better than that for MSANet’s 6.25 and significantly better than that for P2PNet-Soy’s 11.20, indicating better accuracy in seed localization for DeepLabv3+. The overall better balance in precision, recall, and F1 score for MSANet demonstrates that it maintains a certain level of detection capability, whereas P2PNet-Soy performs inadequately, particularly evident in the localization metrics, and DeepLabv3+ shows limitations in recall despite its strong MED performance.

Looking forward, the potential to extend our model to real-time video analysis opens up exciting possibilities for in-field soybean phenotypic data collection. Using a 360° camera for this purpose underscores the potential of our model to open exciting new potential for soybean breeding by enabling true real-time detection and tracking of soybean seeds in their natural environment.

### Error analysis

**Fig. 12.**
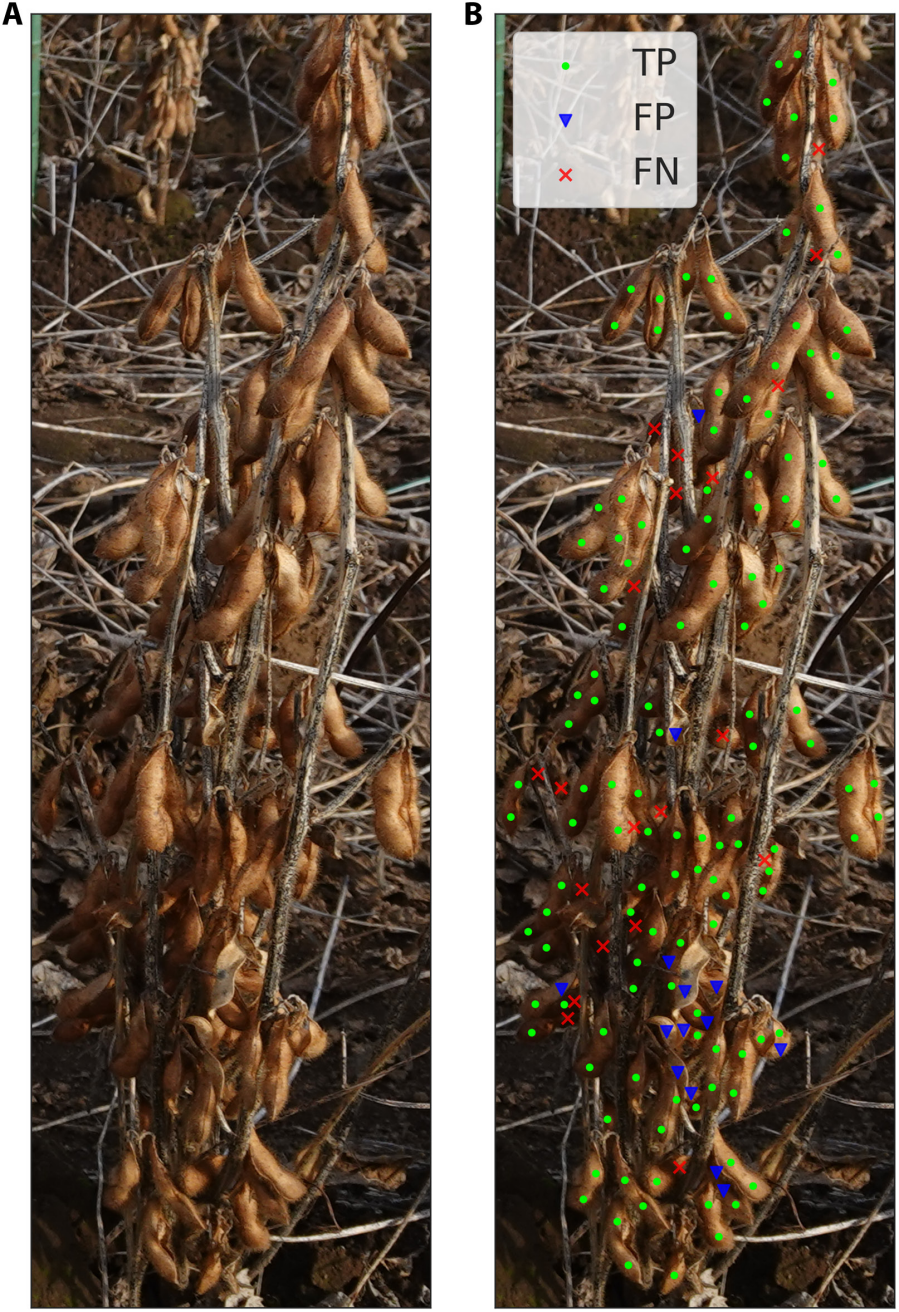
The visualization of the detection result for an individual soybean plant. (A) Raw input image for convenient comparison. (B) MSANet prediction result by categorizing outcomes into true positives (TPs), false positives (FPs), and false negatives (FNs). TP: accurately detected and correctly matched prediction points; FP: instances where the model misidentifies the background as a soybean seed; FN: missed detections of actual soybean seeds.

A performance analysis was conducted to identify shortcomings and opportunities to improve the MSANet model. Evaluation consisted of categorizing outcomes into TPs, FPs, and FNs. Specifically, TPs are accurately detected and correctly matched prediction points; FPs represent instances where the model misidentifies the background as a soybean seed; and FNs denote missed detections of actual soybean seeds. Figure 12 offers a visual depiction of these classification outcomes for a particular soybean plant, and statistical breakdown of detection result types is also provided.

In Fig. 12B, TPs are indicated by green points, demonstrating that the MSANet model successfully detected a substantial number of soybean seeds. This highlights the model’s ability to correctly identify and classify the target objects in most cases. However, the presence of blue points representing FPs indicates instances where the model incorrectly identified background elements as soybean seeds. This suggests that the model could benefit from improved background differentiation techniques to reduce such errors. Additionally, the red crosses representing FNs show that some soybean seeds were missed by the model, indicating a need for enhanced sensitivity in detection algorithms. The diagrams help substantiate the conclusion that while the MSANet model performs well in many cases, there are specific areas—such as reducing FPs and improving detection sensitivity—where performance improvements can be made.

Observations reveal that, although our model proficiently identifies the majority of soybean seeds, a nonnegligible number of FPs and FNs persist. To gain a comprehensive understanding of the model’s error typology, we systematically analyzed images of all soybean plants in the 2022 Dataset, in which a total of 35,565 successfully matched detection points (TPs) were found, along with 9,270 FPs and 6,539 FNs.

This error typology underlines the strengths of our model in reliably detecting soybean seeds while also spotlighting areas for improvement. Despite the model’s high accuracy in identifying soybean seeds, the presence of FPs and FNs suggests ways for further enhancements. Adjustments aimed at reducing these errors could significantly augment the model’s precision, thereby providing a more robust tool for distinguishing between soybean genotypes—a critical step forward in streamlining the soybean breeding process.

### Vertical seed distribution for individual plants

In this study, we used MSANet’s prediction of the vertical distribution of soybean seeds as a novel trait to simplify the breeding process. Firstly, MSANet’s reliability to predict this distribution was validated by comparing its results with the ground truth. Secondly, we predicted the vertical distribution of soybean seeds across 25 soybean genotypes from the 2022 Dataset, demonstrating the potential of this new trait to accelerate soybean breeding.

Traditionally, soybean breeding has emphasized the importance of branching architecture because it influences seed distribution, competition with neighboring plants, and the plant’s fruit-bearing capacity. Complexity and labor-intensive evaluations of multiple traits by phenotyping branching number, angle, pods per branch, etc., hindered understanding relationships between plant structure and seed productivity, and eventually genetic improvement of plant structure. In contrast, our proposed new trait focuses on the vertical seed distribution of soybean plant, a single trait derived from the KDE curves of soybean seeds’ longitudinal (*y*-axis) positions. This novel approach not only simplifies the breeding process but also has the potential on distinguishing between soybean genotypes.

**Fig. 13.**
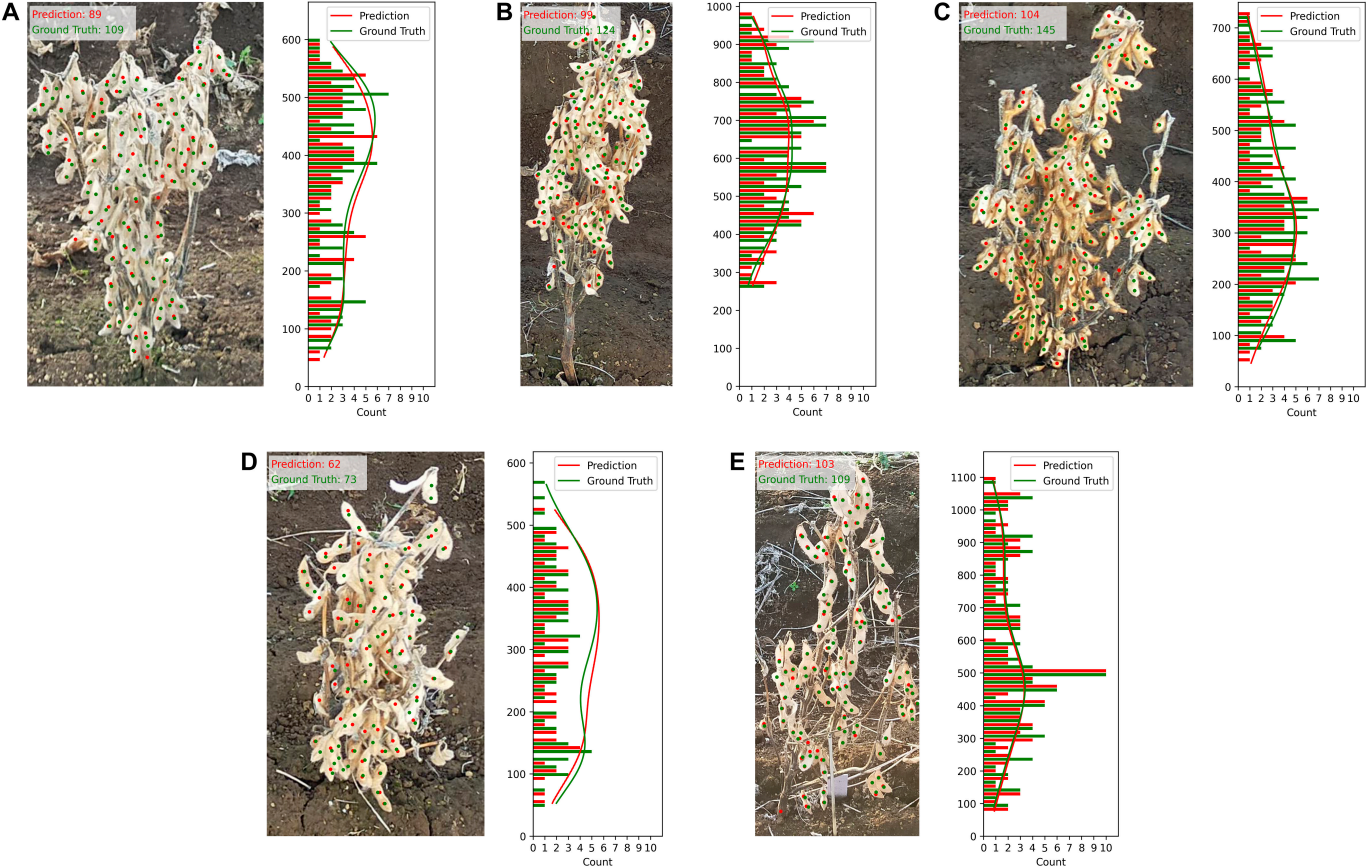
Seed distribution visualization for five individual soybean plant genotypes: (A) *O14E*, (B) *N25E*, (C) *S25E*, (D) *N24E*, and (E) *M307*. The left panels illustrate the seed positions on each plant, while the right panels, with plant height in pixels on the y-axis and seed counts along with KDE values on the x-axis, show the vertical seed distribution. Green represents the ground truth, and red indicates MSANet predictions. Notably, genotype N25E (B) exhibits a higher concentration of seeds in the upper sections compared to S25E (C), suggesting potentially lower seed yield in the lower parts during mechanical harvesting for N25E.

In a detailed examination, the KDE curves derived from the MSANet’s predictions are placed side by side with those obtained from manually annotated ground truths. Figure 13 shows this comparison, showcasing the vertical distribution. of soybean seeds across five soybean genotype: *O14E*, *N25E*, *S25E*, *N24E*, and *M307*. The visual alignment between the model-predicted KDE curves and the ground truth is notable, highlighting the accuracy of our model in capturing the unique vertical distribution patterns for each genotype. In particular, the comparison between (b)N25E and (c)S25E demonstrates distinct differences, with N25E showing a greater concentration of seeds in the upper sections of the plant, which may lead to fewer unharvested seeds in the lower parts during mechanical harvesting. To quantify the differences between the ground truth and the predicted KDE curves, we calculated the Kullback-Leibler (KL) divergence, which ranged from a low of 7.73 x 10^−5^ (*S25E*) to a high of 0.027 (*N24E*) for the five genotypes, all significantly below 1. These results further validate the precision and reliability of MSANet in accurately modeling the vertical seed distribution in soybean plants.

Furthermore, our approach highlights intergenotype distinctions, such as the compact architecture of tendency of (d) *N24E* and seed localization tending toward the top of the short plant, while the more upright and open architecture of (e) *M307* shows denser seed concentration in the lower region (Fig. 13).

### Average vertical seed distribution across genotypes

**Fig. 14.**
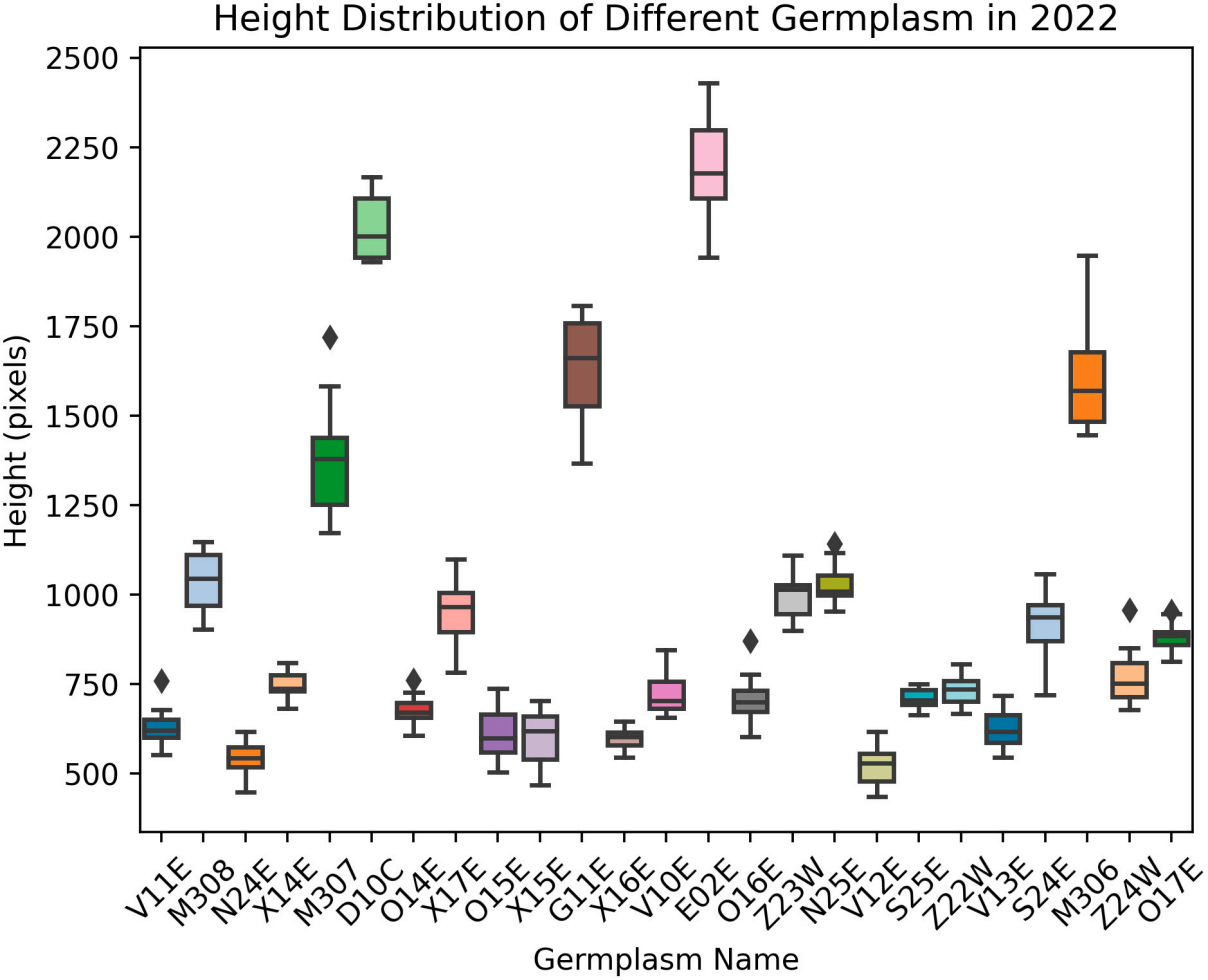
Visualization of the height distribution of different soybean genotypes in dataset 2022, highlighting the substantial variation in plant height across genotypes.

Through the analysis in the previous section, we discovered that there are significant variations in the height of soybean plants even within the same season across different genotypes. Figure 14 clearly illustrates these differences. Furthermore, the soybean lines used in the field experiments were recombinant inbred lines (RILs) derived from parents with extremely different shoot architectures. While typical soybean varieties are notable for their genetic similarity, we specifically chose to study this highly divergent population to maximize variation for training and testing. This strategic choice underscores the importance of genetic diversity in our research.

**Fig. 15.**
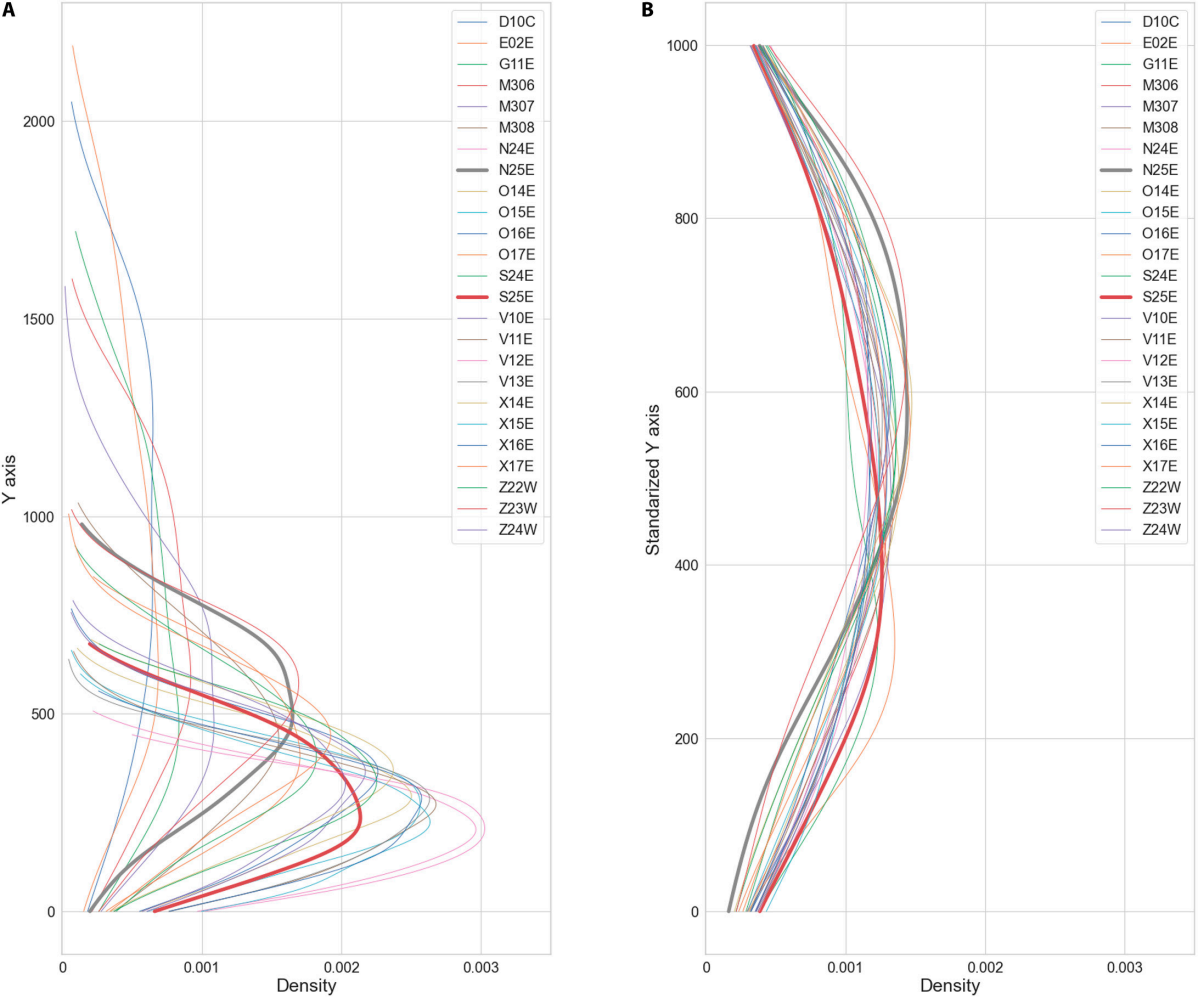
Vertical seed distribution patterns of 25 soybean genotypes were determined by calculating average KDE, applied to the Enlarged 2022 Dataset. (A) Vertical seed distribution, with *y* axis representing the height of the soybean plants in pixels as captured in the original images, allowing for a direct comparison of vertical seed distribution among genotypes. (B) Vertical seed distribution with normalized plant height on the *y* axis, facilitating standardized comparison of vertical seed distribution across different genotypes. *N25E* (Fig. 13B) and *S25E* (Fig. 13C) are shown with bold lines.

Building on this observation, we proceeded to calculate the average KDE curves for samples of different soybean genotypes, aiming to create a representative average KDE curve that captures the typical seed distribution for each genotype. As shown in Fig. 15, vertical seed distribution patterns of 25 soybean genotypes were calculated through our average KDE calculation, based on the prediction of MSANet on the Enlarged 2022 Dataset. Specifically, Fig. 15A shows the distribution, with *y* axis representing the actual height in pixels of the soybean plants as captured in the original images, allowing for a direct comparison of vertical seed distribution across different genotypes. Figure 15B illustrates the KDE curve for each individual plant within the same genotype, followed by summing up and normalization of these KDE values (scaling the *y* axis to a normalized 0 to 1,000 range), facilitating standardized comparison of vertical seed distribution across different genotypes.

Several notable patterns can be observed from the figure. For instance, the genotypes *N25E* and *S25E* exhibit distinctly different seed distribution patterns. In Fig. 15A, the height distribution of these genotypes differs substantially, with *N25E* showing a higher concentration of seeds in the upper region of the plant, whereas *S25E* has a a higher concentration of seeds in the lower region of the plant.

When we analyze Fig. 15B, these inherent differences in the seed distribution patterns become even more apparent. By normalizing the plant height, we remove the influence of the actual plant size, allowing for a clearer comparison of the seed distribution characteristics. The standardized KDE curves show that *N25E* maintains a more concentration distribution in the upper regionalong the vertical axis, while *S25E* exhibits an opposite concentration of seeds in the lower region.

These observations suggest that **N25E* and *S25E** have fundamentally different structural traits, which could be leveraged in breeding programs. *N25E*’s concentrated seed distribution could reduce seed loss in the lower parts during mechanical harvesting compared to S25E.

By analyzing these patterns, we gain valuable insights into the phenotypic diversity among different genotypes, reinforcing the importance of using a diverse set of materials in our breeding studies. This approach not only enhances our understanding of soybean seed characteristics but also aids in identifying genotypes with desirable traits for specific breeding objectives.

### Ablation study

Table 5 presents the results of the ablation study. The table lists the *R*^2^ values, MAE, RMSE, MED, precision, recall, and F1 score for each configuration. The percentages in parentheses indicate the change in performance metrics relative to the full MSANet model, where negative values denote a decrease in performance and positive values denote an increase. Each row in the table corresponds to a different stage being blocked from the MSANet model. The “1/32 stage” row indicates the performance metrics when the smallest 1/32 stage is blocked. The “1/16 stage” row shows the results when both the 1/32 and 1/16 stages are blocked. Similarly, the “1/8 stage” and “1/4 stage” rows present the results when additional stages are blocked sequentially. This hierarchical blocking helps to isolate the impact of each stage on the model’s overall performance.

**Table 5.** Ablation study results

	Counts			Localization			
	*R*^2^↑	MAE↓	RMSE↓	$\bar{\mathrm{MED}}↓$	Precision↑	Recall↑	F1 score↑
**MSANet**	0.87	11.77	16.35	6.22	0.85	0.85	0.85
1/32 stage	0.85 (−2.3%)	12.90 (+9.6%)	18.04 (+10.3%)	6.05 (−2.7%)	0.84 (−1.2%)	0.85 (0.0%)	0.84 (−1.2%)
1/16 stage	0.44 (−49.4%)	90.03 (+664.9%)	118.36 (+623.9%)	7.01 (+12.7%)	0.58 (−31.8%)	0.89 (+4.7%)	0.70 (−17.6%)
1/8 stage	0.35 (−59.8%)	119.75 (+917.4%)	156.98 (+860.1%)	7.98 (+28.3%)	0.51 (−40.0%)	0.88 (+3.5%)	0.65 (−23.5%)
1/4 stage	0.35 (−59.8%)	114.80 (+875.4%)	148.79 (+810.0%)	8.00 (+28.6%)	0.50 (−41.2%)	0.85 (0.0%)	0.63 (−25.9%)

The ablation study results indicate that blocking different stages of MSANet has varying impacts on performance.

Blocking the 1/32 stage shows a slight decrease in performance, with *R*^2^ only decreasing by 2.3%, but MAE and RMSE increase by 9.6% and 10.3%, respectively. This suggests that while *R*^2^ remains relatively stable, there is already a trend of increasing error in MAE and RMSE.

When the 1/16 stage is blocked, the performance degradation becomes more significant. The *R*^2^ value drops sharply by 49.4%, and both MAE and RMSE increase dramatically by 664.9% and 623.9%, respectively. Precision decreases notably, although recall remains high, indicating a substantial drop in the model’s ability to accurately detect soybean seeds while maintaining sensitivity.

Blocking the 1/8 stage results in further performance degradation. Compared to the 1/16 stage, *R*^2^ decreases by an additional 20.5%, with MAE and RMSE continuing to rise. Precision further decreases, and the overall detection accuracy drops significantly, highlighting the critical role of the 1/8 stage in the detection process.

Finally, blocking the 1/4 stage has the most severe impact on performance, with *R*^2^ remaining the same as the 1/16 stage but with further declines in MED, precision, and recall. MED increases by 28.6%, precision drops by 41.2%, and recall falls sharply, resulting in the lowest F1 score among all stages.

These results suggest that while all stages contribute to the model’s performance, the 1/4 and 1/8 stages are particularly critical. The significant performance degradation when these stages are blocked underscores their importance in ensuring accurate and robust seed detection. In contrast, the 1/16 and 1/32 stages, while still important, play a less critical role in comparison.

## Discussion

This study introduced MSANet, a comprehensive deep learning framework for the accurate detection, counting, and localization of soybean seeds. MSANet has demonstrated better performance in identifying soybean seeds across various datasets, significantly outperforming the previous established P2PNet-Soy in both seed counting and localization tasks. The detailed evaluation of MSANet across different datasets affirmed not only its robustness but also its adaptability to diverse phenotypic characteristics inherent in different soybean genotypes.

Although our method has shown a certain degree of generalizability in different soybean images, we also acknowledge that our method is not yet performed optimally in the 360° video images. This demonstrates that for images further away from the camera, our MSANet still has difficulties in recognizing the soybean pods. This may have to do with the lower resolution of the soybean pods in the images in combination with the blurring effects of especially the 1/16× and 1/32× attention layers. For images like these, it may be better to deactivate these last layers to prevent the blurring effect and to allow distinguishable features to be extracted. In line with our ablation study, it is expected that the deactivation of these 2 last attention layers is not going to influence the results to a large extent. This insight can be used in an attempt to further optimize MSANet for more distant images. The same holds for the creation of heatmaps, which might be an adjustable parameter specific to the dataset.

In our study, MSANet reached counting RMSEs between 13 and 18. Compared to similar research in soybean [34,35], we notice that our method is underperforming. These studies were conducted on a smaller and less diverse dataset, with the soybean pods randomly spread on a piece of black cloth such that the pods did not overlap or touch each other, making it easier to obtain better counting results. Given our evaluation on real-field images, our method has been more thoroughly tested in practically relevant conditions, thus providing a better insight into its feasibility for counting and localizing soybean seeds in the field.

We also recognize that counting tasks for objects with different sizes, such as rice panicles, wheat heads, and maize tassels, are equally important for plant breeding [18–20,36,37]. These specific counting tasks demonstrate unique challenges due to the smaller seed sizes or different shapes. To address the challenge of counting smaller objects, MSANet should incorporate a smaller dilation radius, as the current dilation radius was tailored for soybean seed sizes. Adopting a smaller radius would be more appropriate for crops with smaller seeds. Additionally, it may be necessary to adjust the number of attention layers depending on the image scale, such as those captured by drones or ground-based robots. This adjustment could prevent the blurring effects seen with the 1/16× and 1/32× attention layers, allowing for the extraction of more distinguishable features in distant images.

Future work should focus on developing specialized algorithms to detect deflated soybean pods, potentially by incorporating additional training data specifically annotated for this feature. Another potential future direction is to explore the integration of multispectral or hyperspectral imaging, which may provide more detailed information about plant health and phenotypic traits, thereby further enhancing the model’s performance in complex agricultural settings. These enhancements will not only improve MSANet’s utility in soybean breeding but also extend its applicability to other crops, thus offering a versatile tool for counting and localizing objects in the agricultural domain.

## Conclusion

This study introduced a comprehensive deep learning framework for the accurate counting and localization of soybean seeds, a critical task in the enhancement of soybean breeding and genetic research. Our MSANet has demonstrated better performance in identifying soybean seeds across various datasets, significantly outperforming the previous P2PNet-Soy model in both seed counting and localization tasks. The meticulous evaluation of our model across different datasets affirmed not only its robustness but also its adaptability to diverse phenotypic characteristics inherent in different soybean genotypes.

In conclusion, our study underscores the transformative potential of deep learning in agricultural research, providing a powerful tool for soybean phenotyping that combines high accuracy, efficiency, and adaptability. By advancing the capabilities for detailed plant analysis, we contribute to accelerating progress in crop breeding and genetic research, aiming to meet the growing global demand for food production in a sustainable and innovative manner.

## Data Availability

The dataset utilized in this study will be accessible through the following link: https://github.com/UTokyo-FieldPhenomics-Lab/MSANet.
